# Effectiveness of Artificial Intelligence Technologies in Cancer Treatment for Older Adults: A Systematic Review

**DOI:** 10.3390/jcm13174979

**Published:** 2024-08-23

**Authors:** Doris C. Obimba, Charlene Esteva, Eurika N. Nzouatcham Tsicheu, Roger Wong

**Affiliations:** 1 Department of Public Health and Preventive Medicine, Norton College of Medicine, SUNY Upstate Medical University, Syracuse, NY 13210, USA; 2 Department of Geriatrics, SUNY Upstate Medical University, Syracuse, NY 13210, USA

**Keywords:** aging, artificial intelligence, cancer, geriatrics, healthcare, machine learning, older adults, stereotactic body radiotherapy, systematic review, treatment

## Abstract

**Background:** Aging is a multifaceted process that may lead to an increased risk of developing cancer. Artificial intelligence (AI) applications in clinical cancer research may optimize cancer treatments, improve patient care, and minimize risks, prompting AI to receive high levels of attention in clinical medicine. This systematic review aims to synthesize current articles about the effectiveness of artificial intelligence in cancer treatments for older adults. **Methods**: We conducted a systematic review by searching CINAHL, PsycINFO, and MEDLINE via EBSCO. We also conducted forward and backward hand searching for a comprehensive search. Eligible studies included a study population of older adults (60 and older) with cancer, used AI technology to treat cancer, and were published in a peer-reviewed journal in English. This study was registered on PROSPERO (CRD42024529270). **Results**: This systematic review identified seven articles focusing on lung, breast, and gastrointestinal cancers. They were predominantly conducted in the USA (42.9%), with others from India, China, and Germany. The measures of overall and progression-free survival, local control, and treatment plan concordance suggested that AI interventions were equally or less effective than standard care in treating older adult cancer patients. **Conclusions:** Despite promising initial findings, the utility of AI technologies in cancer treatment for older adults remains in its early stages, as further developments are necessary to enhance accuracy, consistency, and reliability for broader clinical use.

## 1. Introduction

Cancer results from uncontrollable cell growth that can affect various organs throughout the body [1]. There are many types of cancer associated with several risk factors, such as sedentary lifestyles, unhealthy diets, and alcohol consumption [2,3]. Amongst these various contributing factors, aging remains among the most impactful and unmodifiable.

Cancer affects everyone, regardless of demographic; however, older adults exhibit a higher risk of cancer diagnosis or cancer-related death [4,5]. The incidence rate for individuals aged 60 years and older is around 1000 per 100,000, significantly higher than the rates of 350 cases and 25 cases per 100,000 for individuals aged 40–49 years old and under the age of 20 years old, respectively. This higher cancer incidence rate among older adults can be attributed to the accumulation of cell damage and weakened or altered immune systems with age. According to the National Cancer Institute (NCI) Surveillance, Epidemiology, and End Results (SEER) Program, half of all cancer cases occur in people over 66 years of age [6].

With the older American adult population projected to reach 72.1 million by 2030 [7], the total number of cancer cases is expected to rise. Although cancer-related deaths have decreased over the past three decades, cancer has remained one of the top leading causes of death [8], making it crucial to explore and address cancer treatment effectively. However, current cancer treatments are typically expensive. In 2015, cancer treatment costs in the U.S. amounted to approximately $183 billion (about $560 per person), with projections estimating a rise to $246 billion (about $760 per person in the U.S.) by 2030 [9]. For older adults, this cost often increases due to the additional need for caregiver support, raising concerns about treatment accessibility [10,11].

Cancer treatments are medical strategies and interventions used to cure, control, or stop cancer’s development and progression [12]. Common treatments include radiotherapy, surgery, immunotherapy, and chemotherapy, with varying effectiveness depending on cancer type, stage, and patient characteristics [13,14,15]. Each treatment carries potential side effects, such as organ damage, hair loss, blood clots, the promotion of metastasis, and possible complications from surgery. Older adult populations are particularly susceptible to these side effects [16,17,18,19,20,21,22]. Efforts are ongoing to develop safer and more effective treatments, but older adults are often underrepresented in research, despite being a group that would greatly benefit [23].

As awareness and excitement around artificial intelligence (AI) technology grows, researchers are exploring ways to integrate AI benefits into clinical medicine to improve healthcare. There is a growing sense that AI has the potential to revolutionize medical practice [24] by assisting physicians, reducing medical errors, improving patient participation, and bridging gaps in care [25,26,27,28,29,30,31,32]. AI is broadly defined as the “use of computers and technology to simulate intelligent behavior and critical thinking comparable to a human being” [27]. It includes two subtypes: virtual AI, which involves neural network-based guidance in treatment decisions, and physical AI, which includes robots assisting in performing surgeries [27,33]. AI’s ability to integrate heterogeneous data makes it a valuable tool for addressing the complexity and multimodality of cancer in the older adult population [5,34,35].

The aim of our review is to examine the effectiveness of AI-incorporated treatments across various studies. Different studies included in this review may use different primary outcomes to measure effectiveness, including overall survival (OS) rate, treatment plan concordance, local control (LC), and progression-free survival (PFS). The OS rate is the percentage of patients surviving over a period after receiving treatment [36]. Treatment plan concordance measures how similar the AI-generated treatment plans are to plans created by multidisciplinary tumor boards (MTBs). Local control (LC), which refers to the complete or partial regression of a tumor compared with its initial volume [37], and progression-free survival (PFS) are the length of time after treatment that a patient goes without their disease worsening [38]. By evaluating outcomes such as these, our review aims to provide a comprehensive assessment of the effectiveness of AI-incorporated cancer treatments.

## 2. Materials and Methods

We developed this systematic review following the Cochrane Handbook and adhered to the Preferred Reporting Items for Systematic Reviews and Meta-Analyses (PRISMA) guidelines to ensure a transparent and reproducible screening process. Studies eligible for inclusion were those that focused on older adults (≥60 years), utilized artificial intelligence in cancer treatments, and were published in English-language, peer-reviewed journals. Exclusion criteria included studies involving participants younger than 60 years, those focusing on diseases other than cancer, studies not using AI technology for cancer treatment, non-English publications, and non-peer-reviewed journals.

Our general search terms included: artificial intelligence, AI, cancer, treatment, older adults, and elderly. In February 2024, three authors separately searched one of three electronic databases: CINAHL, PsycINFO, and MEDLINE via EBSCO. Complete details of the search strategy are listed in Appendix A. Authors imported all references into EndNote and removed duplicates by comparing the first author, year of publication, title, journal, and volume of the papers. Additionally, two authors (D.C.O. and E.N.N.T.) performed forward and backward hand-searching in May 2024 through MEDLINE via PubMed for a more comprehensive search. All authors conducted screenings using Rayyan.

We created our data extraction instrument based on the Cochrane Handbook data extraction item checklist. For each paper, we recorded details such as the first author, year of publication, country of the study, study objective, study design, study duration, sample size, masking techniques, participant allocation, the number and types of interventions, primary outcome and how often it was measured, relevant quantitative results, main conclusions, study limitations, disclosures, funding sources, conflicts of interest, and other relevant information. Concerning the participant characteristics, reviewers also extracted participants’ ages, the percentage of females, race, the type and stage of cancer, and any comorbidities mentioned.

We divided all the studies into three groups and assigned them to pairs of reviewers (C.E. and E.N.N.T., E.N.N.T. and D.C.O., or C.E. and D.C.O.). Each reviewer independently conducted title/abstract and full-text screening and data extraction for their assigned articles. Conflicts for screening and data extraction were resolved by the reviewer not assigned to the paper. This study was registered on PROSPERO (#CRD42024529270).

To determine the effectiveness of the AI interventions, we planned to evaluate how well those interventions performed according to the parameters of success set up by the authors of each study. This means we used the results that researchers obtained from measuring their primary outcomes as indicators of how effective the AI interventions were.

## 3. Results

### 3.1. Study Selection

**Figure 1:**
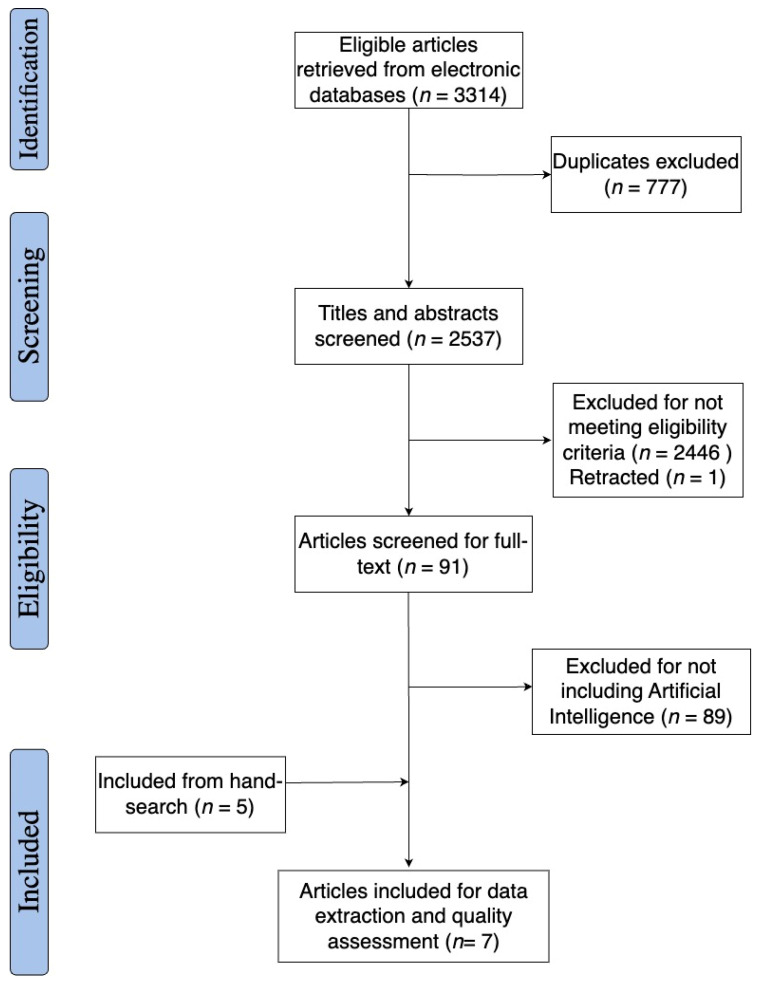
PRISMA flow diagram of our study selection.

Figure 1 provides detailed information on the articles that were included and excluded from this review. We found studies across the three databases and removed 777 duplicates. We removed 2446 ineligible studies and one retracted article through title/abstract screening. After full-text screening, we removed 89 studies for not including artificial intelligence in their interventions. This resulted in two eligible articles for data extraction and quality assessment. Through forward and backward hand searching on the articles, we identified five additional articles that met the study inclusion criteria. The final sample included seven articles.

### 3.2. Characteristics of Included Studies

Among the seven eligible studies, the lowest mean age of participants was 52 years. Some results were stratified by age, allowing us to focus on outcomes for patients aged 60 years and older. The percentage of female participants ranged from approximately 9.1% to 100%. Artificial intelligence interventions were used for the creation of treatment plans (*n* = 3, 42.9%) and stereotactic body radiotherapy (SBRT) (*n* = 4, 57.1%).

The studies focused on cancers of the lung (*n* = 5, 71.4%), breast (*n* = 1, 14.3%), and gastrointestinal system, excluding colorectal cancer (*n* = 1, 14.3%). They included patients with various stages of cancer: all four stages (*n* = 2, 28.6%), stage I (*n* = 3, 42.9%), stages I and II (*n* = 1, 14.3%), or an unspecified stage of cancer (*n* = 1, 14.3%). All seven studies were retrospective. About 42.9% of the studies were conducted in the US, with one study each (14.3%) conducted in India, China, South Korea, and Germany. The characteristics of each included study are more thoroughly listed in Table 1.

The appropriate outcome for each study depended on the AI intervention being used and the study’s purpose. Consequently, we grouped papers with similar outcomes and interventions together when we evaluated the results.

### 3.3. Effectiveness of the AI Interventions in Treatment Planning

Out of our sample of articles, 42.9% (*n* = 3) used either ChatGPT or IBM Watson for Oncology (WFO) to create appropriate treatment plans for patients with lung, breast, or gastrointestinal cancers [39,43,44]. ChatGPT is an AI language model that is trained to understand and appropriately respond to human conversation. Watson for Oncology, on the other hand, is a clinical decision support system trained to create treatment plans from test cases. For each cancer case, it generates treatment plans that it places in one of four categories: recommended, for consideration, not recommended, or not available. While it seems more suitable for this task than ChatGPT, WFO cannot create treatment plans for cases that it is unfamiliar with. Across these studies, researchers selected cancer cases from among those that had previously been reviewed by a multidisciplinary tumor board and manually entered their information into ChatGPT and WFO to create treatment plans. They assessed treatment plan concordance between the AI-generated plans and the MTB. If the WFO’s recommended plans were similar to those of the MTB, they were considered to be concordant. Treatment plans created by ChatGPT that were similar to the MTB’s recommendations were considered concordant. Additionally, researchers for the ChatGPT study determined if the AI-generated plans were geneal recommendations or specific to a patient.

In the two WFO studies (versions 18.4 and 16.4, respectively), concordance ranged from 73% to 93% among participants of all ages [43,44]. However, odds ratios of 0.007 (*p* < 0.001) for adults 75+ years versus <45 years, 0.07 (*p* < 0.001) for adults 65–74 years versus <45 years, and 0.897 (*p* < 0.589) for adults > 60 years versus <60 years indicated that this concordance might decrease for older adults compared with younger age groups. For older adults, WFO could be less likely to generate treatment plans comparable to those made by an MTB. The remaining article focusing on ChatGPT 3.5 compared the plans generated for patients >80 years to plans created for those <80 years. Researchers found that there was a statistically insignificant difference (*p* = 0.2) in the percentage of general and specific treatment plans devised for each age group [39]. ChatGPT 3.5 created specific treatment plans 70% of the time and general plans 30% of the time for those older than 80 years. These respective percentages for those younger than 80 years were 83.2% and 16.8%. Among the case-specific plans, researchers also found an overall treatment plan concordance rate of 83% for all age groups.

### 3.4. Effectiveness of AI Interventions in Stereotactic Body Radiotherapy

The remaining four studies used the AI technology Cyberknife™ (version unspecified) to perform SBRT on patients with lung cancer [40,41,42,45]. For the most part, the operating teams across these studies used X-ray imaging to place fiducial markers on or near patients’ tumors to keep track of them. They carried out CT scans for treatment planning. During the SBRT operation, patients lay supinely on a treatment table while their tumors were treated with radiation through Cyberknife™, with median doses ranging from 40 to 60 Gy. The treatment was administered at least once a day over a period ranging from two to 13 days in the studies. The median biological effective doses (BED) were 105.6 Gy and 132 Gy in three of the studies. [41,42,45]

The 1-year overall survival rates of patients who received Cyberknife™ SBRT in three of these studies were 70% [42], 86% [41], and 96% [45], while the 1-year local control rates in two of these papers were 80% and 100% [42,45]. They also reported median progression-free survival lengths of 19 months, 31 months, and 48 months [41,42,45]. In the remaining article, patients had a 3-year OS rate of 75% and a 3-year locoregional control (LRC) rate of 91% [40]. The objectives, outcomes, and conclusion of each study are more thoroughly listed in Table 2.

## 4. Discussion

The studies in this review show that AI advancements are being made in the performance of SBRT and the development of cancer treatment plans. As evidenced by the reported overall survival (OS) and local control (LC) rates, progression-free survival (PFS) lengths, and treatment plan concordance, these advancements are currently either equally or less effective than standard cancer treatments.

### 4.1. Implications of the AI Treatments

A multidisciplinary tumor board (MTB) convenes healthcare professionals involved in cancer care across various disciplines to collaboratively design evidence-based interventions [46]. This collaborative approach includes physician specialists, nurses, social workers, and mental health professionals, aiming to enhance communication, document diseases accurately, expedite treatment, mitigate risks, and elevate patient satisfaction [46,47]. Importantly, MTBs also alleviate physician burnout and emotional stress [47]. While AI technology holds promise for replicating MTB outcomes, recent studies on WFO and ChatGPT 3.5 suggest it falls short, particularly for older adults. Drawbacks noted in these studies include AI’s difficulty in generating specific treatment plans and adapting to evolving treatment regimens and guidelines [43].

Stereotactic body radiotherapy, typically administered over one to eight treatments using dedicated or nondedicated linear accelerators for the radiation beams, is commonly used on smaller cancers, making it a suitable treatment for lung cancer [48,49]. It is a preferred non-surgical treatment for inoperable lung cancers and older adults seeking to avoid surgical risks. Studies by Watanabe et al. and Shu et al. on SBRT outcomes in older adult patients (≥75 and ≥80 years, respectively) reported encouraging 1-year OS rates ranging from 88.9% to 92.6%, PFS rates exceeding 80% (83.5%), and LC rates of 98.4% and 95.6% [50,51]. Similarly, Aoki et al. and Dong et al. reported favorable 3-year OS rates with SBRT 76% and 81.8%, respectively [52,53]. The OS and LC rates recorded in the Cyberknife™ studies we reviewed are generally comparable to those in the aforementioned papers. However, the wide variation of LC and OS rates between the reviewed studies allude to a need for more papers studying the effectiveness of Cyberknife™ on older adults.

Some AI algorithms demonstrate high effectiveness in predicting cancer diagnoses among older adults and projecting treatment responses, side effects, and complications by leveraging patient-specific data [54]. This capability suggests AI’s potential to significantly enhance health outcomes for older adult cancer patients by leveraging multifaceted patient assessments, including survival, prognosis, and cancer progression predictions using pathology slides, blood biomarkers, and radiologic images [55,56]. Reports indicate greater achievements in improving cancer treatment outcomes and overall health prospects for older adults using AI technologies [54,57].

As AI diagnostic tools in clinical cancer research continue to evolve, they will likely become more effective over time. Our review identifies key AI applications in older adult populations (or older adult patients with cancer) involving survival, prediction, local control (disease stabilization), and treatment plans. Despite promising initial findings, the utility of AI technologies in cancer treatment remains in its early stages, as further developments are necessary to enhance accuracy, consistency, and reliability for broader clinical use.

### 4.2. Gaps in Knowledge

The available research on AI technologies, including machine learning and deep learning algorithms, has shown that these tools can effectively utilize electronic health data to predict clinical parameters [25]. A significant aspect of AI technology is deep learning, which focuses on pattern recognition through deep neural networks (DNNs). These networks assist in interpreting medical scans, pathology slides, vital signs, and more [25].

To name a few applications, DNNs have been used in medical scans to identify lung nodules [58], liver masses [59], pancreatic cancer [60], mammography [61,62], and magnetic resonance imaging [63]. AI technology such as CyberKnife™ SBRT (or robotic SBRT) has proven to be safe and useful for medically inoperable older adult patients with stage I lung cancer [45] and early-stage non-small cell lung cancer (NSCLC) [42].

Moreover, the involvement of AI systems like WFO and ChatGPT in the creation of treatment plans is also being considered. However, studies suggest that it remains uncertain whether these AI systems can replicate treatment plans and partake in clinical decision-making like tumor boards [39].

Our review of AI technologies utilized in cancer treatment among the older adult population found three major gaps. First, AI technologies currently lack a holistic, preventive approach, as their ability to utilize an individual patient’s data is not well developed. Consequently, the predictive capabilities of AI in the healthcare setting remain poorly understood due to the lack of research incorporating robust statistical methodologies and analyses within both studies and real-world clinical environments [25]. Second, the analysis of genomic biological datasets presents an ongoing obstacle for machines and deep learning models. They face difficulties when analyzing somatic cancer mutations [64], RNA sequencing data [65], methylation [66], and single cells [67].

Third, there is a lack of research on specific sub-age groups within the older adult population, leading to a lack of diversity and minimal accessibility to future cancer treatment plans. The use of AI tools can significantly expand the understanding and treatment of cancer by employing transfer-learning algorithms on multiregional tumor-sequencing data [68] and utilizing machine vision to analyze live cancer cells at single-cell resolution [69]. Furthermore, before implementing an algorithm in patient care, the inclusion of minorities in datasets must be attained. It is well known that low socioeconomic status is a significant risk factor contributing to premature mortality, exacerbating the gap in health outcomes [70]. As such, it is imperative to ensure a greater and more accurate representation of the population, recognizing that certain cancers may disproportionately affect specific races and ethnicities.

### 4.3. Future Research and Practice

Our review offers four recommendations for future research and practice. First, investigators should consider implementing variability and diversity in evidence-based studies. This includes greater transparency in study design notation, the adoption of robust methodologies such as randomized controlled trials, and expanding studies to encompass diverse cancer types and stages. These modifications could enhance participation among older adults with cancer.

Second, the careful use and explanation of AI technologies across different racial and ethnic groups should be prioritized. Effective communication and cultural competency are crucial to ensuring engagement in such studies.

Third, there should be a comprehensive assessment of complex medical cases and patient histories among older adult cancer patients. Understanding these factors is essential for interpreting research findings and applying AI technologies effectively.

Lastly, global funding initiatives are necessary to increase visibility and awareness of AI technologies. This can foster greater interest, support, and advancements in AI applications for cancer treatment.

### 4.4. Strengths and Limitations

To the best of our knowledge, this review is the first to focus on the effectiveness of AI in the treatment of older adult cancer patients. While we found a review that briefly touched on using AI to personalize cancer treatment plans for an aging population [54], and a few reviews focused on how AI is used to aid older adults and other age groups with diabetes [71,72], our review is distinct in its specific focus on an older adult population with cancer. Our comprehensive search strategy allowed us to gather the most relevant articles on this topic.

However, the age restrictions applied to our study population resulted in a small sample of studies. Even without these age restrictions, our sample size was likely to be limited since the excitement about AI seems to have outpaced the actual scientific evidence, particularly in healthcare.

Due to the characteristics of the populations in the studies included and the variation among the studies themselves, this review’s results cannot be applied to patients with different types and stages of cancer or to age groups younger than 60.

## 5. Conclusions

AI technologies for cancer treatment among older adult patients present limited research on their potential utility for future implementation within the healthcare sector. There is substantial promise for accelerating medical advancements using AI among patient populations, potentially reducing errors, inefficiencies, and costs associated with doctor–patient interactions [25]. As evidenced by a few retrospective studies, deep learning of digitized pathology slides, for instance, has the potential to improve accuracy and speed of interpretation. [73,74]. The first prospective study to test the algorithmic accuracy of digital pathology slides was performed in a real clinical setting involving the identification of the presence of breast cancer micro metastases in slides by six pathologists compared with a DNN [60]. It was found that combining pathologists and algorithmic interpretations resulted in the best accuracy, with the algorithm reducing the amount of time spent reviewing the slides [75]. These notable advancements emphasize the critical value of a synergistic physician–AI relationship. Our findings indicate an urgent need for AI improvements in cancer survival, prediction, local control (disease stabilization), and treatment plans among the older adult populations in conjunction with robust research studies and data. As AI becomes more mainstream in medicine, further reviews and meta-analyses will be needed to quantify the effectiveness of these interventions and assess the quality of these studies. Further research is required as it will aid in patient assessments, including survival, prognosis, and cancer progression predictions.

## Figures and Tables

**Table 1:** Characteristics of studies included.

Study	Country	AI Used	Age (Percent Female)	Cancer (Stage)	Race and Ethnicity	Study Design
Aghamaliyev et al., 2024 [39]	Germany	ChatGPT 3.5	NR (44.3%)	Gastrointestinal cancer (NR)	NR	Retrospective
Chen et al., 2012 [40]	USA	Cyberknife™ (version unspecified)	Range: 63–87 y (60.0%)	NSCLC (I)	82.5% Caucasian 17.5% African	Retrospective
Ji et al., 2024 [41]	China	Cyberknife™ (version unspecified)	Range: ≥65 y (9.1%)	NSCLC (I, II)	NR	Retrospective
Karam et al., 2013 [42]	USA	Cyberknife™ (version unspecified)	Range: 65–90 (39.0%)	NSCLC (I)	NR	Retrospective
Kim et al., 2020 [43]	South Korea	IBM WFO (version 18.4)	Median: 71 y (16.1%)	Lung Cancer (I, II, III, IV)	NR	Retrospective
Somashekhar et al., 2018 [44]	India	IBM WFO (version 16.4)	Mean: 52 (100.0%)	Breast cancer (I, II, III, IV)	NR	Retrospective
Wang et al., 2017 [45]	USA	Cyberknife™ (version unspecified)	Range: ≥75 y (20.0%)	Lung cancer (I)	NR	Retrospective

Abbreviations: ChatGPT, Chat Generative Pre-Trained Transformer; IBM WFO, International Business Machines Corporation Watson for Oncology; NR, not reported; NSCLC, Non-small cell lung cancer; USA, United States of America.

**Table 2:** Objectives, outcomes, and conclusions of each study included.

Study	Objectives	Outcomes	Conclusions
Aghamaliyev et al., 2023 [39]	ChatGPT 3.5 was used to create appropriate treatment plans for patients with gastrointestinal cancers.	ChatGPT 3.5 created specific treatment plans 70% of the time and general plans 30% of the time for those older than 80 years. These respective percentages for those younger than 80 years were 83.2% and 16.8%, respectively.	When comparing patients older than 80 years to those younger than 80 years, there was a statistically insignificant difference (*p* = 0.2) in the percentage of general and specific treatment plans devised for each age group.
Chen et al., 2012 [40]	CyberKnife (version unspecified) was used on medically inoperable older adult patients with stage I lung cancer and early stage (NSCLC).	Patients had a 3-year OS rate of 75% and a 3-year locoregional control rate of 91%.	CyberKnife is a reasonable treatment option for patients with clinical stage I NSCLC who are not eligible for segmentectomy or lobectomy. This is evidenced through CyberKnife treatment tumor tracking, which resulted in locoregional control of 70% and overall survival of 80% for high-risk surgical patients with clinical stage I NSCLC.
Ji et al., 2024 [41]	Cyberknife SBRT (version unspecified) was used to treat older adult patients with stage I-II central NSCLC.	The 1-year overall survival rate of patients who received these treatments was 86%. The studies reported median progression-free survival lengths of 19 months.	Cyberknife SBRT can safely and effectively control local tumor progression and acceptable radiation toxicity among older patients with centrally located stage I–II NSCLC.
Karam et al., 2013 [42]	Cyberknife SBRT (version unspecified) was used to treat older adult patients with inoperable early stage NSCLC.	The 1-year overall survival rate of patients who received these treatments was 70%. The studies reported median progression-free survival lengths of 31 months, while the 1-year local control rate was 80%.	Cyberknife SBRT can safely and effectively treat patients with inoperable early stage NSCLC. The median biologically effective dose, histology, and tumor size are predictors of local control, whereas tumor size and gender predict overall survival.
Kim et al., 2020 [43]	The use of WFO (version 18.4) was assessed for clinical treatment for patients with lung cancer.	Between the MTB and WFO in patients with lung cancer, there was an overall concordance rate of 92.4%.	Though concordance varied by stage, the treatment decisions of the WFO exhibited a high degree of agreement with those of the MTB.
Somashekhar et al., 2018 [44]	The use of WFO (version 16.4) was assessed for clinical treatment for patients with breast cancer.	Between the MTB and WFO in patients with breast cancer, there was an overall concordance rate of 73–93%.	Though concordance varied by stage, the treatment decisions of the WFO exhibited a high degree of agreement with those of the MTB.
Wang et al., 2017 [45]	Cyberknife SBRT (version unspecified) was used to treat older adult patients with presumed primary stage I lung cancer.	The 1-year overall survival rates of patients who received these treatments was 96%. The studies reported median progression-free survival lengths of 48 months, while the 1-year local control rate was 100%.	Cyberknife SBRT can safely and effectively treat patients with presumed primary stage I lung cancer, especially when there is difficulty confirming pathological malignancy.

Abbreviations: Non-small cell lung cancer (NSCLC), Stereotactic body radiotherapy (SBRT), Multidisciplinary tumor board (MTB), Watson for Oncology (WFO).

## Data Availability

No new data were created or analyzed in this study. Data sharing is not applicable to this article.

## Appendix A. Detailed Search Strategy

**CINAHL**

(“artificial intelligence*” OR AI*) AND (cancer*) AND (treatment*) AND (“older adults*” OR elderly*)

**PsycINFO**

(“artificial intelligence” OR “artificial intelligences” OR AI*) AND (cancer*) AND (treatment*) AND (“older adults” OR elderly*)

**MEDLINE via EBSCO**

(“artificial intelligence*” OR AI*) AND (cancer*) AND (treatment*) AND (“older adults*” OR elderly*)
